# Genetic testing including targeted gene panel in a diverse clinical population of children with autism spectrum disorder: Findings and implications

**DOI:** 10.1002/mgg3.354

**Published:** 2017-12-21

**Authors:** Louisa Kalsner, Jennifer Twachtman‐Bassett, Kristin Tokarski, Christine Stanley, Thyde Dumont‐Mathieu, Justin Cotney, Stormy Chamberlain

**Affiliations:** ^1^ Connecticut Children's Medical Center Farmington CT USA; ^2^ University of Connecticut School of Medicine Farmington CT USA; ^3^ Courtagen Life Sciences Woburn MA USA

**Keywords:** autism spectrum disorder, *KIRREL3*, microarray, racial/ethnic diversity, targeted gene panel, *TSC2*

## Abstract

**Background:**

Genetic testing of children with autism spectrum disorder (ASD) is now standard in the clinical setting, with American College of Medical Genetics and Genomics (ACMGG) guidelines recommending microarray for all children, fragile X testing for boys and additional gene sequencing, including PTEN and MECP2, in appropriate patients. Increasingly, testing utilizing high throughput sequencing, including gene panels and whole exome sequencing, are offered as well.

**Methods:**

We performed genetic testing including microarray, fragile X testing and targeted gene panel, consistently sequencing 161 genes associated with ASD risk, in a clinical population of 100 well characterized children with ASD. Frequency of rare variants identified in individual genes was compared with that reported in the Exome Aggregation Consortium (ExAC) database.

**Results:**

We did not diagnose any conditions with complete penetrance for ASD; however, copy number variants believed to contribute to ASD risk were identified in 12%. Eleven children were found to have likely pathogenic variants on gene panel, yet, after careful analysis, none was considered likely causative of disease. *KIRREL3* variants were identified in 6.7% of children compared to 2% in ExAC, suggesting a potential role for *KIRREL3* variants in ASD risk. Children with *KIRREL3* variants more often had minor facial dysmorphism and intellectual disability. We also observed an increase in rare variants in *TSC2*. However, analysis of variant data from the Simons Simplex Collection indicated that rare variants in *TSC2* occur more commonly in specific racial/ethnic groups, which are more prevalent in our population than in the ExAC database.

**Conclusion:**

The yield of genetic testing including microarray, fragile X (boys) and targeted gene panel was 12%. Gene panel did not increase diagnostic yield; however, we found an increase in rare variants in *KIRREL3*. Our findings reinforce the need for racial/ethnic diversity in large‐scale genomic databases used to identify variants that contribute to disease risk.

## 1 INTRODUCTION

The heritability of autism spectrum disorder (ASD) is supported by recent studies demonstrating a recurrence risk for ASD of 11%–19% in families with one affected child compared to 1%–2% risk for ASD in the larger US population (Constantino, Zhang, Frazier, Abbacchi, & Law, 2010; Ozonoff et al., 2011). Twin studies further support the critical importance of genetics to risk for ASD, revealing concordance rates for ASD as high as 88%–95% in monozygotic twins, compared to 31% in dizygotic twins (Rosenberg et al., 2009; Taniai, Nishiyama, Miyachi, Imaeda, & Sumi, 2008). Despite these data, our ability to identify the critical genetic factors impacting risk in any one individual remains limited. Numerous studies utilizing chromosomal microarray (CMA), targeted gene sequencing, whole exome sequencing (WES) and, more recently, whole genome sequencing have identified hundreds of genes associated with autism risk (Levy et al., 2011; O'Roak et al., 2012; Sanders et al., 2011, 2012; Stessman et al., 2017; Yuen et al., 2017), yet genetic factors contributing to ASD risk are varied and complex. They include single gene disorders, copy number variants, and inherited and de novo rare (present in <1% of the population) and common sequence variants (present in >1%). At present, we have only a limited understanding of the role played by these variants in disease causation, particularly those inherited from seemingly unaffected parents.

Despite, these challenges, it remains important to pursue genetic testing when evaluating a child with ASD. Understanding the precise etiology of ASD in an individual can provide critical information to families by helping direct medical care to identify and treat comorbidities known to occur with a specific disorder; eliminate further, unnecessary diagnostic testing; better define the risk of recurrence; enable attainment of services; and, in rare cases, may even allow for targeted treatment of ASD symptoms. Current guidelines from the ACMG recommend microarray for all children with ASD without a recognizable genetic diagnosis and fragile X testing for boys (Schaefer & Mendelsohn, 2013). In addition, single gene sequencing is recommended including *MECP2* for girls and *PTEN* for those with macrocephaly. Consideration of metabolic screening, brain MRI and X‐linked disability gene panel is also recommended where medical history, physical exam and/or family history support it. ACMG practice guidelines suggest this approach is estimated to yield a diagnosis in 30%–40% of individuals with ASD.

As testing utilizing high throughput sequencing becomes increasingly available, it is important to consider the role of this testing modality in the clinical setting. A recent study from Newfoundland and Labrador, Canada found that WES and CMA contribute to the identification of a molecular diagnosis in 8% and 9% of children with ASD respectively (Tammimies et al., 2015). In addition to WES, multi‐gene panels via next generation sequencing (NGS) are increasingly available for the clinical evaluation of children with ASD, some containing as many as 2000 genes (GeneDx, Gaithersburg autism/ID panel). Targeted gene panels have some advantages over WES; they are typically less costly and often provide higher average gene coverage. As the list of genes associated with ASD risk grows, and the cost of NGS falls, it may become more logical to screen a large panel of genes when evaluating an individual with ASD, rather than sequencing a series of single genes as recommended in the ACMG guidelines.

Despite the tremendous diagnostic opportunity provided by NGS, whether utilizing gene panel or WES, genetic testing is not without its challenges, as interpretation of results can be difficult due to the frequent identification of variants of uncertain significance (VUS). It is increasingly recognized that genetic variants identified and reported as potentially associated with disease often are interpreted as such based on frequency of the individual variant in large‐scale databases, along with predictive models which consider the potential impact of the variant on function of the gene. However, variants deemed rare based on frequency in large‐scale genomic databases, may, in fact, be fairly common in specific racial/ethnic groups (Manrai et al., 2016). This discrepancy in variant frequencies can lead to misinterpretation of genetic results, with “rare” variants often reported as potentially pathogenic, when in fact, they are more likely benign and unrelated to disease, as evidenced by their frequency in healthy sub‐groups of the population.

Herein, we studied a clinical population of 100 children diagnosed with ASD at Connecticut Children's Medical Center (CCMC) using microarray and fragile X testing, as well as next generation sequencing of a panel of genes associated with risk for ASD and other neurodevelopmental disorders. The panel utilized was a commercial test available from 2014 to 2016 (devSEEK ‐ Courtagen Diagnostics Laboratory, Woburn) designed to include a set of genes with the most support for clinical utility and with literature supporting association with ASD and/or developmental disability. Our goals were to evaluate the yield of this model of genetic testing in a socio‐demographically diverse clinical population, to explore the role of rare variants as contributors to ASD risk, and to identify phenotypic features that may be associated with specific genetic findings in our study population. Lastly, given the diversity of the patient population served by CCMC, we sought to consider the impact of racial/ethnic background on the interpretation of genetic results emerging from targeted gene sequencing.

## 2 MATERIALS AND METHODS

Ethical Compliance: This study was approved by the institutional review board (IRB) at Connecticut Children's Medical Center.

As the only free‐standing Children's hospital in Connecticut, Connecticut Children's Medical Center (CCMC) serves children and families from across the state; the patient population is representative of the state's socio‐demographic diversity. The study sample included 100 consecutive children evaluated in the CCMC Autism Neurogenetics Program, age 21 months to 17 years, who met eligibility requirements and for whom informed consent was obtained from a parent or legal guardian. Referrals to neurogenetics came primarily from the Autism Spectrum Assessment Program (ASAP), a diagnostic clinic in which children are assessed by a developmental‐behavioral pediatrician and speech‐language pathologist. Additional children were referred from the Department of Neurology where they were seen for evaluation and management of ASD and associated symptoms.

All children enrolled in the study (i) had a confirmed diagnosis of ASD by a developmental‐behavioral pediatrician or child neurologist experienced in ASD, (ii) scored in the full autism range on the Autism Diagnostic Observation Schedule, Second Edition (ADOS‐2) (Lord et al., 2012) and (iii) either met Diagnostic and Statistical Manual of Mental Disorders, 5th Edition (DSM‐5; American Psychiatric Association, 2013) criteria for ASD or scored in the autism range on the Childhood Autism Rating Scale‐2 (CARS‐2) (Schopler, Van Bourgondien, Wellman, & Love, 2010). Children with a known genetic diagnosis suspected to be causative of ASD were excluded. These included one child with a 1 Mb deletion at 1p31.1–1p31.2, one individual with Down syndrome, and a child with a 264 kb deletion at 7q11.22, including three exons of the AUTS2 gene. In addition, children with a history of brain injury or prematurity below 32 weeks were excluded. Socio‐demographic information was collected and family and medical histories were reviewed for all participants; each underwent a thorough history and physical examination with attention to dysmorphic features by a clinical geneticist (LK). A parent or guardian completed questionnaires including Vineland‐II (Sparrow, Balla, Cicchetti, & Doll, 2005), PDDBI (Cohen & Sudhalter, 2005), and Social Responsiveness Scale (SRS) (Constantino & Gruber, 2012). Each child underwent cognitive assessment using either the Mullen Scales of Early Learning (Mullen, 1995) or the Stanford–Binet Intelligence Scales, Fifth Edition (Roid, 2003). Participants had a blood sample sent for microarray analysis and fragile X testing for 73 of the 76 males enrolled. Three high functioning males did not have fragile X testing for the following reasons: one child was found to have XXY; a second child's parents had been screened prenatally for fragile X and had repeat numbers in the normal range; because of the high out of pocket predicted cost for testing, the third child's parents chose not to pursue the diagnosis. Both microarray and fragile X testing were performed at one of several clinical laboratories chosen based on insurance and physician preference as part of routine recommended clinical care. The microarray platforms varied with copy number variant (CNV) detection reported to range from 5 Kb at one lab to >200 Kb at another, which provided additional detection of 5–200 Kb CNV's in 200 targeted regions. CNVs were classified as pathogenic, variant of uncertain significance (VUS) likely pathogenic, unspecified or likely benign, or benign by the clinical testing laboratory. Fragile X testing was performed using DNA amplification of the FMR1 gene by polymerase chain reaction, with laboratory policy to reflex to either southern blot analysis or methylation PCR analysis for those in the premutation or full mutation range. Parent testing was done, when possible, to clarify inheritance pattern of copy number variants identified by microarray. Unfortunately, this was not possible for many families, often due to limitations in insurance coverage. Separate blood samples were sent to Courtagen Diagnostics Laboratory (Woburn, MA), for targeted exon sequencing using a commercially available panel containing a select set of genes that have well established association with developmental delay, intellectual disability, or ASD or emerging evidence of disease association. Genes were selected using Agilent's HaloFlex capture probes and sequenced on Illumina MiSeq insruments. The devSEEK panels (v2‐v9) contained between 101 and 237 genes. The gene panel was continually updated by Courtagen with genes added based on data emerging in the literature. Determination of the pathogenicity of variants was based upon a combination of factors, including variant population frequency, predicted in silico pathogenicity and, when available, inheritance pattern. The pathogenicity calls were made by the diagnostic testing laboratory with each variant scored on a 5‐point scale by Courtagen in a manner consistent with ACMG recommendations (Richards et al., 2015). In total, 100 children underwent sequencing of a minimal set of 79 genes, while 90 had a larger panel of 161 consistently sequenced genes (see Tables S1 and S2 for lists). Parent testing using saliva samples was performed whenever possible, if recommended by the genetic testing laboratory, typically in cases where clarification of the inheritance pattern of a variant of uncertain significance might help to determine its pathogenicity. In addition, parent testing was completed for those children in whom rare *TSC2* and *KIRREL3* variants were identified.

To determine population frequency of rare variants in the Exome Aggregation Consortium (ExAC) data, the data were retrieved from Version 0.3.1 (Lek et al., 2016). The ExAC database contains pooled data from over 60,000 individuals collected from numerous large scale exome sequencing projects. The population frequency was calculated for the entire population as well as subgroups for each gene individually. Variants present in more than 1% of the whole ExAC population were excluded from the calculations.

To compare *TSC2* and *KIRREL3* variant frequencies in a larger ASD cohort, we retrieved whole exome sequence variant call data for individuals sequenced from the Simons Simplex Collection by Sanders et al. (2012) and deposited in the National Database for Autism Research (NDAR). These variants were first converted from hg18 to hg19 coordinates using LiftoverVcf from Picard Tools (v 1.14) and filtered based on quality metrics originally published by Sanders et al. (2012). Resulting variant call format files were then converted for annotation with ANNOVAR (Wang, Li, & Hakonarson, 2010) and overlaid with frequency data from 1,000 Genomes (Auton et al., 2015) and ExAC. All rare *TSC2* and *KIRREL3* variants for probands, siblings, and parents were then extracted. Where appropriate, variants were grouped by family identification number to determine de novo versus inherited variation and pattern of inheritance.

## 3 RESULTS

Of the 100 children enrolled, 76 were male and 24 were female (3.2:1 male: female). Age range of participants was 21 months to 17 years with mean age at diagnosis and enrollment of 37 months (3.1 years) and 57 months (4.5 years), respectively (Table 1). Ninety‐six families provided information on race/ethnicity and four declined. Of the 96 families for whom information was available, 50 (52%) described the child as White with no racial/ethnic minority group identification. Forty‐six families (48%) identified at least in part with a racial/ethnic minority group as follows: 7% African American, 5% Asian, 15% Hispanic/Latino and 21% more than one racial/ethnic group. When including those who self‐identified with more than one racial/ethnic minority group, the enrolled children's ancestry included the following racial/ethnic backgrounds: 18% African American, 26% Hispanic/Latino, 9% Asian and 2% Native American. When compared to the statistics released by the US Census Bureau (US Census Quickfacts V2016) our study population included fewer children identified as White or African American, while a substantially greater number self‐identified as “more than one race/ethnicity.” This resulted in a greater representation of children with identified African American, Hispanic/Latino and Asian ancestry (Table 2).

**Table 1:** Demographics

Gender	76 male, 24 female (3.2:1)
Age at enrollment	
Mean	57 months
Median	51 months
Mode	32 months
Range	21 months to 17 years
Median age at ASD diagnosis	32 months

**Table 2:** Racial/ethnic background

	Study population self‐reported	Percent identifying in part with each group	Percent in US 2016 census	Percent in ExAC database
African American	7	18	13.3	8.3
Asian	5	9	5.7	20.0
Caucasian (not Latino)	52	68	61.3	60.4
Hispanic/Latino	14.5	26	17.8	9.5
More than one race/ethnicity	21		2.6	Not reported

Cognitive assessment was attempted in 98 of 100 children enrolled and was successful in 86, with 12 children refusing or not responsive to testing. Of the 86 children completing the assessment, 22 (26%) scored in the average or above average range (>80), 8 (9%) were borderline (70–79), 28 (33%) were mildly impaired (55–69) and 28 (33%) were moderately impaired (40–54). The ADOS‐2 is a standardized, play‐based assessment used to gather information in the autism diagnostic process; a 10‐point scale of “Comparison Scores” is used to standardize autism severity across modules of the ADOS‐2. All children underwent the ADOS‐2, administered by trained personnel familiar with the clinical evaluation of children with ASD. Participants receiving a score of 6 or higher (designating the “full autism range” on the ADOS‐2) were included in the study. Scores are available for 98 children enrolled in the study, with the overall severity range being verified for the two participants for whom an exact score was not available. Fifty‐four percent of participants scored in the more affected range (9–10) as compared to 46% scoring in the mild to moderate range (6–8) (Table 3).

**Table 3:** Cognitive range (combined Mullen and Stamford–Binet scores)

Cognitive range of participants	Number of subjects	Percent
Above average (120 + )	3	3.5
Average (80–119)	19	22
Borderline (70–79)	8	9
Mildly impaired (55–69)	28	32.5
Moderately impaired (40–54)	28	32.5
Total	86	100

### 3.1 CMA

All 100 children underwent clinical microarray testing. Of these, 18 had one or more copy number variants identified (Table 4), while 82 were negative for copy number change. After careful consideration, we interpreted four of the CNVs as likely pathogenic: one each of Klinefelter syndrome (XXY), a 1.8 Mb duplication at 1q21.1–q21.2, a maternally inherited 402 kb deletion at 15q11.2, and a 365 kb duplication at 2p16.3 interrupting the *NRXN1* gene. Two of these variants, 15q11.2 deletion and 2p16.3 duplication, were reported as VUS by the genetic testing company but, as explained in greater detail in the Discussion, we felt there was significant evidence in the literature to support a contributory role of these variants to disease risk. CNVs of uncertain significance were found in eight additional children, including three deletions and five duplications. Six children were found to harbor benign or likely benign CNV's including two deletions and four duplications. The child found to harbor a 1q21.1–1q21.2 duplication also carried a smaller, maternally inherited duplication interpreted as a VUS likely benign. In addition, two children were found to have regions of homozygosity. Excluding variants interpreted by the testing company as likely benign, the yield of microarray in our study population was 12% when including pathogenic CNV's and variants of uncertain significance. This is consistent with a reported yield of approximately 10% in the literature (McGrew, Peters, Crittendon, & Veenstra‐Vanderweele, 2012; Tammimies et al., 2015).

**Table 4:** Results of chromosomal microarray analysis

Copy number variant	Coordinates	Size	Inheritance
Likely pathogenic			
XXY			
1q21.1‐q21.2^a^	Duplication	1.8 Mb	Mother negative; Father U
15q11.2	Deletion	402 Kb	M
2p16.3	Duplication	365 Kb	NA
Variants of uncertain significance			
1q31.1	Deletion	148 Kb	NA
5q23.3	Deletion	383 Kb	NA
12q24.33	Duplication	302 Kb	NA
13q12.11	Duplication	2.2 Kb	NA
7q36.1	Duplication	381 Kb	M
10q23.1	Duplication	1.8 Mb	M
9q33.1	Deletion	157.1 Kb	M
5q35.3	Duplication	365 Kb	NA
Likely benign			
15q14	Duplication	336.2 Kb	U
2q37.3	Duplication	384 Kb	P
3p26.2	Deletion	13.3 Kb	U
7p11.2	Duplication	35.5 Kb	U
2p25.2^a^	Duplication	621.7 Kb	M
2p12	Duplication	1088.7 Kb	U
16p12.2	Deletion	355 Kb	U

NA, Not available; M, Maternal; U, Unknown.

a These variants were found in the same individual.

### 3.2 Fragile X

Fragile X testing was completed on 73 of 76 (96%) males enrolled. Of these, 71 had repeat numbers in the normal range (<45) and two were in the borderline/gray zone, having 49 and 53 repeats. No children were found to have a premutation or fragile X diagnosis.

### 3.3 Gene panel

Among the 100 probands, 11 children were found to have pathogenic or likely pathogenic variants (VUS likely pathogenic) as reported by the genetic testing company (Table 5 and Table S4). Eight of the likely pathogenic variants were found in genes associated with autosomal recessive disease, thus the variants detected suggested only carrier status. None of these individuals was found to have a CNV involving the second allele. Of the remaining three variants, one child was found to harbor a paternally inherited and extremely rare missense variant in *CNTNAP2,* which is predicted to be deleterious. A second child had a splice variant in *CNTN4*, anticipated to result in alternative splicing and consequent deletion of 57 base pairs with resultant frameshift. The third child was found to harbor a rare de novo variant in *TSC2* (c.856A>G). This was interpreted by the testing laboratory as likely associated with disease as it (i) was a de novo change and (ii) occurred in a region of the gene with moderate evolutionary conservation. No mutations were identified in *MECP2* or *PTEN* in our study population.

**Table 5:** Gene panel—laboratory reported likely pathogenic variants

Gene name	Variant	Associated disease	Inheritance	Inheritance pattern
*CNTNAP2*	chr7.hg19.g.146818169G>C	Gilles de la Tourette syndrome, schizophrenia, epilepsy, autism, ADHD, and intellectual disability (polygenic); Pitt‐Hopkins like syndrome 1 (AR); Cortical Dysplasia Focal Epilepsy Syndrome (AR)	Paternal	AR/M/P
*CNTN4*	chr3.hg19.g.3076476T>C	Autism	Paternal	Unknown
*MED23*	chr6.hg19.g.131948602TTC>T	Intellectual disability, autosomal recessive 18	U	AR
*TSC2*	chr16.hg19.g.2105504A>G	Tuberous Sclerosis Complex	De novo	AD
*CUBN*	chr10.hg19.g.16970304T>C	Megaloblastic anemia‐1, Finnish type	U	AR
*VPS13B*	chr8.hg19.g.100833547G>A	Cohen syndrome	Paternal	AR
*DHCR7*	chr11.hg19.g.71146886C>G	Smith–Lemli–Opitz syndrome	U	AR
*PNKP*	chr19.hg19.g.50365022ACGCTACCTGG>A	Early infantile epileptic encephalopathy‐10 (EIEE10)	U	AR
*MKKS*	chr20.hg19.g.10393443CT>C	McKusick–Kaufman syndrome	U	AR
*TRAPPC9*	chr8.hg19.g.140744276G>T	Autosomal recessive intellectual disability	U	AR
*LINS*	chr15.hg19.g.101110181AAAGTC>A	Autosomal recessive intellectual disability 27	U	AR

AR, autosomal recessive; AD, autosomal dominant; M, multifactorial; P, polygenic; U, Unknown.

### 3.4 Rare‐variant frequencies

To determine whether rare missense variants in the genes represented in the devSEEK panel contribute to ASDs, we compared the frequency of rare variants found in each gene with that expected in a normal population, as reported in the ExAC database (Table 6). We noted significantly increased frequencies of rare variants in three genes in our study population: *TSC2, MKKS* and *KIRREL3* (*p* ≤ .05). We found rare *TSC2* variants in 18 of 100 subjects as compared to 9.8% in the ExAC database (*p* = .0062) (Table 7). Among the *TSC2* variants was a de novo missense variant predicted to be pathogenic by the genetic report (described above), as well as an in‐frame insertion not seen in the ExAC population. Rare variants in *KIRREL3* were found in 6 of 90 (6.7%) children in our study population but are reported in only 2.0% in the ExAC database (*p* = .001). Two of the individuals with rare *KIRREL3* variants in our population were siblings and all of these variants were inherited from an unaffected parent. All of the *KIRREL3* variants occurred in regions that are either moderately or highly conserved, and four of the six variants were predicted to be deleterious by two different protein prediction algorithms (Table 8). Rare variants in *MKKS* were noted in 5 of 70 (7.1%) study patients compared to 3.1% in ExAC (*p* = .047) (Table 9). One of the variants in *MKKS*, a frameshift mutation, was predicted to be pathogenic by the genetic report. In addition, we noted a decrease in rare variants in *PCNT* (14% compared to 24% in ExAC), though the significance of this was unclear. Subsequent correction of the *p*‐values for multiple comparisons indicated that variants in *MKKS* were not significantly enriched in our ASD population compared to controls, while variants in *PCNT* were not significantly decreased (Table 9).

**Table 6:** Genes with rare variants frequently identified in our sample

Gene	Number with rare variant	Number tested	ASD frequency	ExAC frequency	Max sub‐population requency	*p* value a<.05	B‐H critical (10% FDR)	Significant after MCC
*KIRREL3* a	6	90	0.067	0.020	0.034	.001	0.005	Yes
*TSC2* a	18	100	0.18	0.098	0.30	.0062	0.0225	Yes
*PCNT* a	13	9	0.14	0.24	0.76	.041	0.02	No
*MKKS* a	5	70	0.071	0.031	0.039	.047	0.0025	No
*CREBBP*	11	100	0.11	0.076	0.027	.20	0.015	No
*RELN*	11	100	0.11	0.15	0.54	.23	0.0125	No
*TRAPPC9*	8	100	0.08	0.055	0.11	.28	0.01	No
*KIF1A*	7	90	0.078	0.055	0.11	.36	0.0075	No
*VPS13B*	19	100	0.19	0.16	0.43	.45	0.005	No
*ANK3*	14	90	0.16	0.18	0.51	.53	0.0025	No

MCC, multiple comparisons correction.

a *p* value (<.05) denotes significant difference between ASD and ExAC frequencies, as measured by chi‐square with 1 degree of freedom. Benjamani–Hochberg critical value (B–H critical) denotes the *p* value necessary to achieve significance after correcting for multiple comparisons.

**Table 7:** *TSC2* variants

Variant	Predictions	Max Sub‐pop frequency	ExAC frequency	Inheritance
chr16.hg19.g.2121617T>C	PP: B; MT: D	0.00572	0.000364	M
chr16.hg19.g.2105504A>G	PP: B; MT: D	0.00018	9.88E‐05	DN
chr16.hg19.g.2134508G>T	PP: B; MT: D	0.025217	0.006683	M
chr16.hg19.g.2122297G>C	PP: D; MT: D	0.000453	0.000248	P
chr16.hg19.g.2138546G>A	PP: B; MT: D	0.009771	0.005086	U
chr16.hg19.g.2138546G>A	PP: B; MT: D	0.009771	0.005086	M
chr16.hg19.g.2137939A>G	PP: B; MT: D	0.000122	3.36E‐05	U
chr16.hg19.g.2120487G>A	PP: D; MT: D	0.005559	0.003308	M
chr16.hg19.g.2121873G>A	PP: B; MT: D	0.000554	0.000249	U
chr16.hg19.g.2138600G>A	PP: D; MT: D	0.001163	0.000168	P
chr16.hg19.g.2110765C>T	PP: B; MT: D	0.002212	0.000944	P
chr16.hg19.g.2112558G>A	PP: B; MT: D	0.002941	0.001535	U
chr16.hg19.g.2134981CCTT>C	Not listed; in frame deletion	0.01842	0.010604	M
chr16.hg19.g.2138320C>CCAGCGGGTAGGGAATATGGGGCTCCCT	Not listed; in frame insertion		Not in ExAC	P
chr16.hg19.g.2120487G>A	PP: D; MT: D	0.005559	0.003308	U
chr16.hg19.g.2134981CCTT>C	Not listed	0.01842	0.010604	U
chr16.hg19.g.2121536G>A	PP: D	0.000927	0.000184	P
chr16.hg19.g.2120487G>A	PP: D; MT: D	0.005559	0.003308	U

PP, Polyphen2; MT, Mutation Taster; B, Benign; D, Deleterious; M, Maternal; P, Paternal; DN, Denovo; U, Unknown.

**Table 8:** *KIRREL3* variants

Variant	Conservation	Predictions	Inheritance
chr11.hg19.g.126314949C>T	High	PP: D; MT: D	M
chr11.hg19.g.126314888G>A	Moderate	PP: D; MT: D	P
chr11.hg19.g.126314895C>G	High	PP: D; MT: D	M
chr11.hg19.g.126314949C>T	High	PP: D; MT: D	M
chr11.hg19.g.126305185C>A	High	PP: E; MT: D	P
chr11.hg19.g.126333139C>A	Moderate	PP: B; MT: D	P

PP, Polyphen2; MT, Mutation Taster; B, Benign; D, Deleterious; E, Equivocal; M, Maternal; P, Paternal.

**Table 9:** *MKKS* variants

Variant	Conservation	Predictions
chr20.hg19.g.10394147C>T	Moderate	PP: D; MT: D
chr20.hg19.g.10393304G>T	Low	PP: B; MT: D
chr20.hg19.g.10389422T>C	Low	PP: B; MT: D
chr20.hg19.g.10389422T>C	Low	PP: B; MT: D
chr20.hg19.g.10393443CT>C	Moderate	Frameshift/pathogenic

PP, Polyphen2; MT, Mutation Taster; B, Benign; D, Deleterious; E, Equivocal.

### 3.5 TSC2

We observed rare *TSC2* variants in our ASD population at almost twice the frequency of the normal population as per the ExAC database. Heterozygous, loss‐of function, pathogenic variants in *TSC2* cause Tuberous Sclerosis Complex (TSC). TSC is characterized by a constellation of clinical findings, including 3 or more hypomelanotic macules, cortical dysplasias, cardiac rhabdomyoma and renal angiomyolipoma. ASD is diagnosed in as many as 50% of children with ASD (Jeste, Sahin, Bolton, Ploubidis, & Humphrey, 2008), therefore, we looked closely for signs of TSC in the 18 children in our population with rare variants in *TSC2*. All 18 children with *TSC2* variants underwent a thorough skin examination with none having 3 or more hypomelanotic macules. MRI of the brain was completed in 7 of the 18 children and none had features suggestive of TSC. Thus, after thorough examination of the children, we found none of them had clinical manifestations of TSC.

At least one prior study suggested that variants in *TSC2* might contribute to nonsyndromic ASD (Schaaf et al., 2011). However, another study seeking to determine whether *TSC2* variants contribute to nonsyndromic autism risk did not see an increased frequency of *TSC2* variants in ASD probands from a cohort of 300 individuals from the Simons Simplex Collection (SSC) (Bahl et al., 2013). Therefore, we tried to replicate our finding in a larger ASD population, analyzing whole exome sequencing results available in NDAR for the Simons Simplex Collection. Of 876 individuals screened by Sanders et al. (2012), 98 had at least one *TSC2* variant that fit rare frequency criteria from ExAC (11.2%). Two probands and one unaffected sibling were found to have single de novo loss of function variants. For 200 families with four members sequenced, we found no difference in the presence of a rare *TSC2* variants in mothers, fathers, probands or siblings. Frequencies of rare *TSC2* variants were 17% (*n* = 34), 10% (*n* = 20), 11.5% (*n* = 23), and 13% (*n* = 26), respectively, revealing no difference in parental origin or significant enrichment in probands.

On closer analysis of the data, we noted that some of the rare variants in *TSC2* were more common in specific racial/ethnic subgroups. Of all individuals reported in the ExAC browser, 9.8% carry at least one rare *TSC2* variant. However, many of these rare *TSC2* variants are found commonly in the African American (AA) subgroup, with one so‐called rare variant occurring in approximately 8% of African Americans surveyed by ExAC. In fact, the overall frequency of “rare” *TSC2* variants in the AA subgroup was 30%. We noted that African Americans are under‐represented in the ExAC browser (8.5%) compared to both the US population (13%) and our study population (18% with some AA ancestry). The apparent increase in rare *TSC2* variants in the initial analysis of our study sample is thus proportional to the higher AA representation in our patient population as compared to ExAC. Thus, our findings reflect substantial population differences in our cohort and SSC relative to large databases such as ExAC and do not implicate *TSC2* as a contributor to risk for ASD, in line with the conclusion reached by Bahl et al. (2013) and Stessman et al. (Stessman et al., 2017).

### 3.6 KIRREL3

We found that the rate of *KIRREL3* variants in our cohort was more than three times the rate found in the ExAC population (*p* = .001). *KIRREL3* has a low haplo‐insufficiency score of 5%, suggesting mutations which result in loss of function of one copy of the gene can be expected to result in disease (Firth et al., 2009; Huang, Lee, Marcotte, & Hurles, 2010), thus we looked more closely at phenotypic features of the children with *KIRREL3* variants (Table 10). Interestingly among the six children with *KIRREL3* variants, minor facial dysmorphism, such as coarsened features, flattened nasal bridge or epicanthal folds, were noted in four. Two children were found to have a Chiari I malformation. Evidence of intellectual disability was noted in all six children with *KIRREL3* variants, with scores below 70 on the cognitive assessment as compared to 61% of the total study group (*p* = .06). Family history of ASD was also more common in the *KIRREL3* group (67% with ASD in a first degree relative as compared to 19% of the total study population, *p* = .05) (Table 11). We also assessed whether the enrichment we observed in our ASD population was replicated in the Simons Simplex Collection. Out of 200 families with four members sequenced, only 11 individuals had at least one *KIRREL3* variant that fit rare frequency criteria from ExAC. One proband and one unaffected sibling were found to have single de novo loss of function variants. The numbers of rare *KIRREL3* variants found in mothers, fathers, probands, and siblings were 2,3,2, and 4, respectively. Overall, the numbers of rare variants in *KIRREL3* are very small and preclude any conclusive analysis for or against replicating our data in this cohort.

**Table 10:** Phenotypic features of *KIRREL3* patients

Patient	Facial features	Medical issues	MRI	Family history	Language function
1	Coarse; large ears; prominent jaw	Anemia treated with iron infusions	Chiari I	Brother ASD^a^ Maternal tracheostomy in childhood, speech delay	Nonverbal
2	Epicanthal folds; Flat nasal bridge	Hives; Allergic enteropathy	ND	Brother with ASD	Nonverbal
3	Epicanthal folds; Flat nasal bridge	None	Chiari I	Brother with ASD	Marked delay
4	Flat nasal bridge; Coarse features	Anemia treated with iron infusions; Central apnea	Normal	Sister ASD^a^ Maternal tracheostomy in childhood, speech delay	Marked delay
5	Non‐dysmorphic	Constipation	ND	Father depression; paternal grandfather schizophrenia	Mild delay
6	Non‐dysmorphic	None	ND	None	Moderate delay

ND, not done.

a Patients 1 and 4 are siblings.

**Table 11:** Comparison of *KIRREL3* patients to ASD cohort

	*KIRREL3* (percent) *N* = 6	Other ASD (percent) *N* = 94	*p* value
Intellectual disability (IQ < 70)	6 (100)	50/82 (61)	.06
ADOS comparison score 9–10	2 (33)	51/92 (55)	NS
Regression	2 (33)	14/79 (18)	NS
Seizures	0	11/90 (12)	NS
Dysmorphic features	4 (67)	33/89 (37)	NS
Family history of ASD (parent/sibling)	4 (67)	18/93 (19)	.02^a^
Family history of psychiatric disease (parent/sibling)	2 (33)	33/93 (35)	NS

NS, not significant.

a Indicates finding is significant.

### 3.7 MKKS

Although ultimately found not to be significant, we also observed an increase in rare variants in *MKKS*, a gene which encodes for a chaperone family protein which acts to stabilize unfolded proteins when exposed to heat shock or stress (Slavotinek & Biesecker, 2001). MKKS specifically acts to stabilize BBS7, which plays a role in ciliary trafficking (Barbelanne, Hossain, Chan, Peranen, & Tsang, 2015). Homozygous mutations in *MKKS* cause Bardet‐Biedl syndrome, which manifests with polydactyly, obesity, retinitis pigmentosa and intellectual disability (Forsythe & Beales, 2003) or Mc‐Kusick Kaufman syndrome, associated with congenital heart disease, genitourinary abnormalities and polydactyly (Slavotinek, 2002). None of these features was present in those with rare variants in our study population. *MKKS* has a high haplo‐insufficiency score of 36% and, like other genes associated with autosomal recessive disease, the presence of rare deleterious or loss of function variants likely suggests only carrier status. Thus, the increase in heterozygous rare variants in *MKKS* in our study population is unlikely to be related to risk for ASD (Firth et al., 2009; Huang et al., 2010).

## 4 DISCUSSION

Genetic testing of children with ASD has become increasingly common with the estimated diagnostic yield of a thorough clinical genetics evaluation suggested to be as high as 30%–40% (Schaefer & Mendelsohn, 2013). Current ACMG guidelines recommend microarray and fragile X for boys, with numerous studies reporting clinically relevant CNVs in 10% of children with ASD and fragile X in 0.5%. In addition, it has been shown that sequencing of *MECP2* is diagnostic in 4% of girls with ASD and *PTEN* in 5% of individuals with macrocephaly and autism. ACMG guidelines also suggest considering metabolic screening and X‐linked disability gene panel in select cases. A variety of gene panels for ASD and intellectual disability are now clinically available. These allow sequencing of many potentially causative genes simultaneously and may be more cost effective than a strategy of testing for multiple individually rare conditions.

To better understand the yield of this approach, we performed genetic testing including microarray, fragile X (males) and a targeted gene panel, consistently sequencing up to 161 genes associated with autism risk, in a well‐characterized clinical population of 100 children with ASD. We did not diagnose any conditions with complete penetrance for ASD and found no cases of fragile X syndrome, but results from microarray analysis suggested a diagnosis in four children (XXY, 15q11.2 del, 1q21.1–q21.2 duplication, duplication at 2p16.3 involving *NRXN1*). Individuals with Klinefelter syndrome, have a variety of cognitive, language and behavioral deficits as well as ASD symptomology (Bruining et al., 2014; Davis et al., 2016). 15q11.2 deletions have been shown to confer a modestly increased risk of ASD, especially upon maternal inheritance (Chaste et al., 2014). 1q21 microduplications are frequently identified in ASD cohorts (Sanders et al., 2015) and a distinct phenotype has been recognized, manifesting with ASD, intellectual disability, macrocephaly and dysmorphic features (Rosenfeld et al., 2012). The 2p16.3 duplication is predicted to disrupt the *NRXN1* gene. De novo deletions of 2p16.3, which include *NRXN1* are repeatedly found in ASD cohorts (Bena et al., 2013). An additional 8 children were found to have copy number variants of uncertain significance. Though the impact of the individual CNVs remains uncertain, one cannot exclude their contribution to ASD risk. Each involved coding regions of one or more genes (see Table S3). The 9q33.1 deletion was initially reported as a likely benign variant but later reclassified by the genetic testing company as a copy number change of uncertain significance in light of a publication reporting enrichment of deletions involving *TRIM32 and ASTN2* in males with neurodevelopmental disorders including ASD, ADHD and anxiety (Lionel et al., 2014). The deletion was passed from an unaffected mother to her son; inasmuch as greater penetrance in males has been observed with ASTN2/TRIM32 deletion, the deletion may play a role in ASD risk in our patient. If we include all CNVs, we considered likely pathogenic and CNVs of uncertain significance, excluding those reported as likely benign, the yield of microarray analysis in our study population was 12%, which is in the range of values reported in numerous other studies (McGrew et al., 2012; Tammimies et al., 2015).

The gene panel utilized in our study, which consistently sequenced at least 161 genes in the majority of those enrolled, yielded 11 likely pathogenic variants. Most of these variants (8/11) occurred in genes known to cause severe disease with an autosomal recessive inheritance pattern, and thus, heterozygous carriers are typically unaffected; however, the contribution of these heterozygous variants to ASD has not been studied and cannot be entirely ruled out. The other three variants (in *CNTNAP2*,* CNTN4*, and *TSC2*) would ordinarily be considered as likely contributing to the ASD diagnosis. Variants in *CNTNAP2* and *CNTN4* have been considered potentially contributory to risk for ASD, Tourette Disorder and psychiatric disease (Alarcon et al., 2008; Bakkaloglu et al., 2008; Roohi et al., 2009; Verkerk et al., 2003), but a recent study in which six *CNTN* and four *CNTNAP* genes (including *CNTN4* and *CNTNAP2*) were subjected to targeted next generation exon sequencing in 2704 ASD cases and 2747 controls did not find any association of rare variants in these genes with ASD (Murdoch et al., 2015). Although the *TSC2* variant reported as likely pathogenic was a de novo variant, we do not consider this variant to be causative of ASD in the individual based on further analysis of *TSC2* variants in our population (discussed below). In summary, we do not think any of these three variants are contributing to ASD.

Thus, although genetic test reports in our population, utilizing combined fragile X, microarray and targeted gene sequencing would suggest an estimated 23% yield for identifying pathogenic genetic aberrations (when including likely pathogenic CNVs, copy number changes of uncertain significance and likely pathogenic or pathogenic variants from gene panel), we would estimate the diagnostic yield to be approximately 12%. The targeted gene panel we utilized did not increase the diagnostic yield in our study population. Of note, we did not identify any individuals with mutations in *MECP2* (including 24 females tested) and no cases with *PTEN* mutation (including five children in our sample with macrocephaly). Our results suggest that the estimated diagnostic yield of 30%–40% as proposed in the ACMG guidelines paper by Schaefer and Mendelsohn (2013) may be higher than can be expected in a typical clinical population of children with ASD, though we did not include results of brain imaging or metabolic studies in our analysis. The yield of targeted gene panels in the evaluation of individuals with ASD has not been well defined. There is one report from 2016 describing a yield of 13.6% for possibly contributory variants from testing of 50 Spanish children utilizing a 44 gene targeted panel (Alvarez‐Mora et al., 2016). Their criteria for defining a variant as relevant was based primarily on its predicted *in silico* pathogenicity. We did not consider *in silico* predictive models sufficient evidence to support a diagnosis, and this likely explains our lower yield. Diagnostic yield from whole exome sequencing appears to be significantly higher than the targeted gene panel we utilized, with 9%–25% of individuals reported to have likely disease causative variants identified using WES (Rossi et al., 2017; Tammimies et al., 2015). The continually expanding list of genes playing a role in ASD risk and ongoing recognition of new, individually rare, disease‐causing genes suggest panels of 1000 or more genes may be more informative in the evaluation of individuals with ASD.

The commercially available gene panel we utilized was updated several times by the testing company to include genes with new evidence to support a role in ASD risk. Several genes which have been consistently found to contribute to ASD risk, such as *CH*D8 (Neale et al., 2012; O'Roak et al., 2012; Talkowski et al., 2012)*,* were not fully assessed in our analysis as they were not included in the gene panel for the majority of our patients. For instance, *CHD8* was only sequenced in 29 of our patients. Our study has several limitations, including a relatively small sample size, lack of parental data for many of the CNVs and variants identified and limitations of the commercially available gene panel including the relatively small number of genes consistently sequenced in our cohort. A higher diagnostic yield might be observed if a larger gene panel was studied; however, the continually expanding list of ASD risk genes makes it difficult to be confident that any gene panel can be “complete”. For this reason, we believe that whole exome sequencing is likely to be more informative and definitive in the identification of mutations contributing to ASD risk in affected individuals. We would thus recommend proceeding to WES for those individuals with ASD who are felt to warrant more extensive genetic evaluation, based on clinical features such as severity of disability, associated congenital anomalies, abnormal head size or presence of associated medical issues such as epilepsy. A recent publication reported results of whole genome sequencing (WGS) in over 2,500 individuals with ASD and identified 18 new candidate genes for ASD, supporting the notion that WGS will allow for even greater diagnostic yield in ASD than WES (Yuen et al., 2017). Though more study of the clinical utility of WGS is needed, it is likely that ultimately it will become the test of choice in the evaluation of individuals with ASD.

Although sequencing of a modest panel of genes associated with ASD risk did not result in the identification of a distinct molecular diagnosis in any children in our study population, we found rare variants in potential ASD candidate genes in most individuals tested. Rare variants are believed to play a major role in risk for autism and can be inherited from unaffected parents (Kosmicki et al., 2017; Yuen et al., 2015). They may act in concert with other rare or common variants to increase risk for ASD. To better understand if the rare variants we identified might play a role in risk for ASD in our population, we compared the rate at which we identified rare variants in individuals with ASD to that found in a normal population using the ExAC database. This allowed us to identify the three genes in which rare variants occurred more frequently in our study group; *TSC2, MKKS* and *KIRREL3*. Ultimately the increase in rare variants in *MKKS* in our ASD cohort was found to be insignificant compared to controls, and was thus dropped from further consideration. However, we were interested in the potential role of *TSC2* and *KIRREL3*.

Most notable was the greater than three times increase in the rate of rare variants in *KIRREL3* in children enrolled in our study as compared to the population in the ExAC database. Because rare variants in *KIRREL3* are not common in any racial/ethnic group (maximum subgroup frequency 3.4%), it seems less likely that racial/ethnic diversity could have affected the interpretation of our results. *KIRREL3* encodes one of a group of synaptic cell adhesion molecules (SCAM), functioning to connect pre and postsynapses during the process of synapse formation and maturation via extracellular adhesion domains and signal transduction through a cytoplasmic tail (Baig, Yanagawa, & Tabuchi, 2017; Liu et al., 2015). Disruption of *KIRREL3* has been reported in patients with neurodevelopmental disorders (Guerin et al., 2012; Talkowski et al., 2012), and de novo mutations have been found in whole genome analysis of twins with ASD (Michaelson et al., 2012). *KIRREL3* interacts at its cytoplasmic tail with CASK, a protein that interacts with many SCAMs and appears to serve an important role in intracellular signaling pathways (Liu et al., 2015). Mutations in *CASK* are known to result in microcephaly with pontocerebellar hypoplasia and severe intellectual disability (Hayashi et al., 2012), further evidence that *KIRREL3* and *CASK* play a critical role in neurodevelopment.

Children with rare *KIRREL3* variants in our study population more often had minor facial dysmorphism noted on examination including coarse features, flattened nasal bridge or epicanthal folds. There is a report in the literature of one individual with severe ID, who had a balanced translocation, interrupting exon of 1 of *KIRREL3* at one of the breakpoints (Bhalla et al., 2008). She had a similar pattern of facial features including flattened mid‐face, thus it is possible that mild facial dysmorphism is a feature seen more commonly in individuals who have *KIRREL3* mutations. Evidence of intellectual disability was noted in all six children with *KIRREL3* variants in our study population, suggesting *KIRREL3* dysfunction may be associated with a more severe pattern of deficits in children with ASD. This is consistent with prior publications associating non‐synonymous *KIRREL3* variants with intellectual disability (Bhalla et al., 2008). Family history of ASD was also more common in the *KIRREL3* group (67% with ASD in a first degree relative as compared to 19% of the total study population, *p* = .05), thus it is possible that *KIRREL3* variants more often play a role in multiplex families. The *KIRREL3* variants we identified were all inherited from an unaffected parent suggesting incomplete or variable penetrance as is often observed in ASD risk genes. However, a family history of autism or psychiatric disease was commonly observed, supporting variable phenotype in those with *KIRREL3* variants, and the likely role of other genetic and environmental factors in the development of ASD in these patients.

One of the most striking observations from our data was the increased frequency of rare *TSC2* variants in our study patients compared to the ExAC database. *TSC2* encodes the tuberin protein, which dimerizes with the *TSC1* product hamartin and acts to inhibit function of AKT/mTOR, a major regulator of neuronal cell growth and proliferation (Han & Sahin, 2011; Laplante & Sabatini, 2012). Tuberin regulates neuronal migration, axon formation and synaptic plasticity, all processes that may be impaired in ASD, supporting the notion that variants could impact neurodevelopment even without systemic features of disease and thus could contribute to risk of nonsyndromic autism. A similar increase in rare, potentially deleterious variants in *TSC2* in an ASD population when compared to controls was noted in a study by Kelleher et al. (2012), suggesting a potential role for *TSC2* in risk for nonsyndromic ASD. However, upon careful analysis of the data we recognized that, while the frequency of rare *TSC2* variants was similar between our cohort and the SSC probands, this frequency was not increased compared to unaffected siblings or parents in this collection. This demonstrated that there is no enrichment for *TSC2* variants in ASD individuals compared to unaffected individuals in a larger ASD cohort.

In addition, we found that the reported racial/ethnic ancestry of our study population as well as the SSC population, differed substantially from the population ancestry that is available in the ExAC database (Table 2). In fact, the reported racial/ethnic background of the ExAC database's sample is not representative of the current US population. There are notable differences in the number of individuals of non‐European ancestry in this database when compared to US population demographics and our clinically derived study cohort. In the case of *TSC2*, variants that appear rare in commonly used databases may in fact be common in subgroups of different ancestry. Careful consideration of the variants detected, the database being used for normative data and the comparative known ancestry of the patient and database cohorts is indicated to avoid reporting dubious associations with ASD. Furthermore, while not specific to ExAC, our findings reinforce the need for a more representative sample of the US population in the databases being used for interpretation of genomic study results if we are to more reliably interpret potentially pathogenic sequence changes in whole exome and genome sequences obtained in the future.

The importance of considering ancestry when interpreting the clinical relevance of rare variants was highlighted in a recent publication by Manrai et al. (2016) in the New England Journal of Medicine. The group reported the misinterpretation of pathogenic variants causative of hypertrophic cardiomyopathy among those of African American ancestry. They noted that variants initially reported as pathogenic were later reclassified as benign, as they were observed commonly in this subgroup and did not have significant association with disease. These false positive reports had important clinical consequences, leading to screening of “at risk” family members, recommendations regarding limitation of activity in carriers and in some cases overestimation of risk in mildly affected individuals leading to consideration of insertion of implantable defibrillators.

While databases such as ExAC have greatly expanded the utility of large sequencing based data sets, they should be used with caution in the clinical and research interpretation of genomic results given their limited representation of the US population. Clinicians and researchers need to be cognizant of the role of an individual's unique ancestry in the accurate interpretation of the relevance of genomic variants. Utilization of high throughput sequencing, including targeted gene panels and whole exome sequencing, is becoming increasingly common in the clinical evaluation of children with ASD. As larger numbers of individuals are evaluated and undergo sequencing, our understanding of the impact of individual variants, including their clinical relevance considering an individual's racial/ethnic background, will likely improve as well.

## DISCLOSURES

Christine Stanley is employed at Courtagen Life Sciences Inc.
