# Supplementary material for: Genetic testing including targeted gene panel in a diverse clinical population of children with autism spectrum disorder: Findings and implications
Source: Mol Genet Genomic Med. 2017 Dec 21;6(2):171–85. doi: 10.1002/mgg3.354 (PMC5902398; doi:10.1002/mgg3.354)
Supplement: Supplementary file 1 [file MGG3-6-171-s001.docx]

Table S1: List of 79 genes across gene panels for 100 patients

| *AFF2* | *EHMT1* | *KIAA2022* | *OPHN1* | *SLC2A1* |
| --- | --- | --- | --- | --- |
| *AP1S2* | *FGD1* | *L1CAM* | *PAFAH1B1* | *SLC9A6* |
| *ARX* | *FMR1* | *MAN1B1* | *PCDH19* | *SMC1A* |
| *ATRX* | *FOLR1* | *MBD5* | *PHF6* | *SMC3* |
| *BCKDK* | *FOXG1* | *MECP2* | *PNKP* | *SRPX2* |
| *BRAF* | *FOXP1* | *MED12* | *PQBP1* | *TBX1* |
| *CACNA1C* | *FOXP2* | *MED23* | *PRSS12* | *TCF4* |
| *CASK* | *GABRB3* | *MEF2C* | *PTCHD1* | *TECR* |
| *CDKL5* | *GRIA3* | *MID1* | *PTEN* | *TRAPPC9* |
| *CHD7* | *GRIK2* | *NHS* | *PTPN11* | *TSC1* |
| *CNTNAP2* | *GRIN2B* | *NIPBL* | *RAB39B* | *TSC2* |
| *CRBN* | *HDAC8* | *NLGN3* | *RAI1* | *TUSC3* |
| *CREBBP* | *HOXA1* | *NLGN4X* | *RELN* | *UBE2A* |
| *DCX* | *HPRT1* | *NRXN1* | *RPS6KA3* | *UBE3A* |
| *DHCR7* | *HRAS* | *NSD1* | *SCN1A* | *VPS13B* |
| *DMD* | *KDM5C* | *NSUN2* | *SHANK3* |  |

Table S2: List of 161 genes across gene panels for 90 patients

| *ACSL4* | *CASK* | *FGD1* | *HUWE1* | *NLGN3* | *PTEN* | *T3GAL3* |  |
| --- | --- | --- | --- | --- | --- | --- | --- |
| *AFF2* | *CC2D1A* | *FGF8* | *IGBP1* | *NLGN4X* | *PTPN11* | *STXBP1* |  |
| *ALG6* | *CCDC22* | *FLNA* | *IL1RAPL1* | *NRXN1* | *RAB39B* | *SYN1* |  |
| *ANK3* | *CDH15* | *FMR1* | *IQSEC2* | *NSD1* | *RAD21* | *SYNGAP1* |  |
| *AP1S2* | *CDKL5* | *FOLR1* | *KCNJ10* | *NSDHL* | *RAI1* | *SYP* |  |
| *AP4B1* | *CHD7* | *FOXG1* | *KDM5C* | *NSUN2* | *RELN* | *TBX1* |  |
| *AP4E1* | *CLIC2* | *FOXP1* | *KIAA2022* | *OCRL* | *RPL10* | *TCF4* |  |
| *AP4M1* | *CNTNAP2* | *FOXP2* | *KIF1A* | *OFD1* | *RPS6KA3* | *TECR* |  |
| *AP4S1* | *CRBN* | *FRMPD4* | *KIRREL3* | *OPHN1* | *SCN1A* | *TRAPPC9* |  |
| *ARFGEF2* | *CREBBP* | *FTSJ1* | *KLF8* | *PACS1* | *SCN2A* | *TSC1* |  |
| *ARHGEF6* | *CTCF* | *GABRB3* | *L1CAM* | *PAFAH1B1* | *SHANK2* | *TSC2* |  |
| *ARHGEF9* | *CTNNB1* | *GATAD2B* | *L2HGDH* | *PAK3* | *SHANK3* | *TSPAN7* |  |
| *ARID1B* | *CUL4B* | *GDI1* | *MAN1B1* | *PCDH19* | *SLC16A2* | *TUBA1A* |  |
| *ARX* | *D2HGDH* | *GRIA3* | *MAOA* | *PCNT* | *SLC25A1* | *TUSC3* |  |
| *ATP6AP2* | *DCX* | *GRIK2* | *MBD5* | *PDHA1* | *SLC2A1* | *UBE2A* |  |
| *ATRX* | *DDHD2* | *GRIN2A* | *MECP2* | *PHF6* | *SLC6A8* | *UBE3A* |  |
| *BCKDK* | *DHCR7* | *GRIN2B* | *MED12* | *PHF8* | *SLC9A6* | *UPF3B* |  |
| *BCOR* | *DLG3* | *HCFC1* | *MED23* | *PLP1* | *SLC9A9* | *VLDLR* |  |
| *BRAF* | *DMD* | *HDAC8* | *MEF2C* | *PNKP* | *SMARCB1* | *VPS13B* |  |
| *BRWD3* | *DYNC1H1* | *HOXA1* | *MID1* | *PQBP1* | *SMC1A* | *ZDHHC15* |  |
| *C12orf57* | *DYRK1A* | *HPRT1* | *NAA10* | *PRPS1* | *SMC3* | *ZDHHC9* |  |
| *CA8* | *EHMT1* | *HRAS* | *NHS* | *PRSS12* | *SMS* | *ZEB2* |  |
| *CACNA1C* | *ERLIN2* | *HSD17B10* | *NIPBL* | *PTCHD1* | *SRPX2* | *ZNF711* |  |
